# Top-Down Control in Contour Grouping

**DOI:** 10.1371/journal.pone.0054085

**Published:** 2013-01-10

**Authors:** Gregor Volberg, Andreas Wutz, Mark W. Greenlee

**Affiliations:** 1 Institut für Psychologie, Universität Regensburg, Regensburg, Germany; 2 Center for Cognitive and Brain Sciences, University of Trento, Rovereto, Italy; University of Leuven, Belgium

## Abstract

Human observers tend to group oriented line segments into full contours if they follow the Gestalt rule of 'good continuation'. It is commonly assumed that contour grouping emerges automatically in early visual cortex. In contrast, recent work in animal models suggests that contour grouping requires learning and thus involves top-down control from higher brain structures. Here we explore mechanisms of top-down control in perceptual grouping by investigating synchronicity within EEG oscillations. Human participants saw two micro-Gabor arrays in a random order, with the task to indicate whether the first (S1) or the second stimulus (S2) contained a contour of collinearly aligned elements. Contour compared to non-contour S1 produced a larger posterior post-stimulus beta power (15–21 Hz). Contour S2 was associated with a pre-stimulus decrease in posterior alpha power (11–12 Hz) and in fronto-posterior theta (4–5 Hz) phase couplings, but not with a post-stimulus increase in beta power. The results indicate that subjects used prior knowledge from S1 processing for S2 contour grouping. Expanding previous work on theta oscillations, we propose that long-range theta synchrony shapes neural responses to perceptual groupings regulating lateral inhibition in early visual cortex.

## Introduction

**Figure 1:**
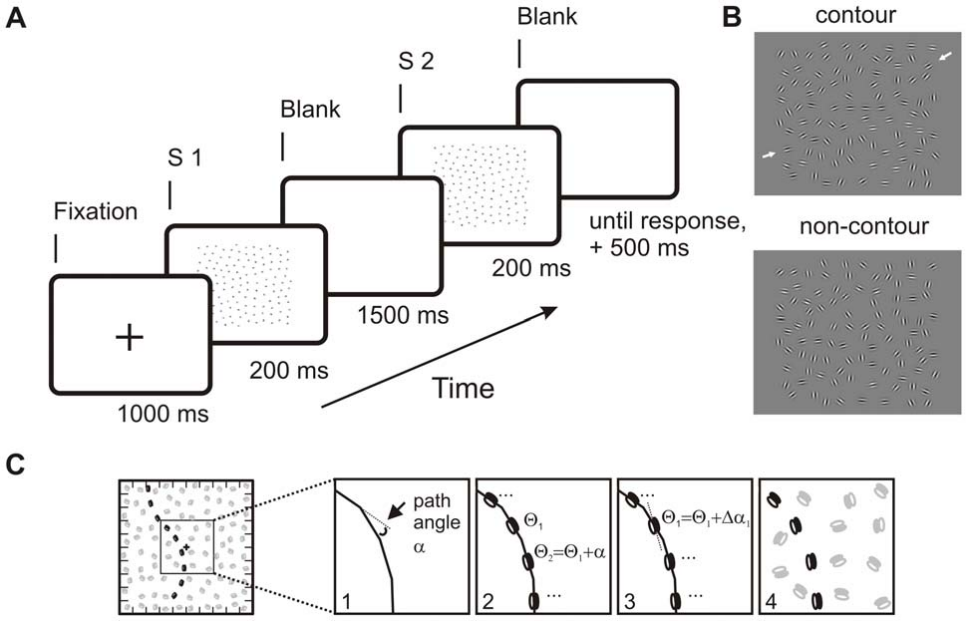
This figure shows the task and the stimuli used in the present study. **A** A typical trial sequence. Pairs of contour and non-contour stimuli were presented in a random order, where the participant indicated whether the first stimulus (S1) or the second stimulus (S2) contained the ‘hidden’ contour. **B** Examples for contour- and non-contour stimuli. White arrows (not shown in the experiment) mark the beginning and the end of the contour. Note that the orientation of the Gabor elements, but not their number or position differ in the contour and non-contour conditions. **C** Illustrates the construction of a contour stimulus. The stimulus array was subdivided into a 10 by 10 grid of possible Gabor element locations. Contours were constructed along traces of invisible line segments, with a 23° angle (α) between adjacent lines (step 1). Gabor elements were placed on the center of each line, collinear to its orientation Φ (step 2). An orientation jitter Δα was added to Φ(step 3), and then empty grid cells were filled with randomly oriented Gabor elements (step 4).

It was already noted by early Gestalt psychologists that the human visual system tends to group local stimulus elements into global wholes. Such grouping is often based on simple rules such as similarity, proximity, or good continuation of the local elements [1]. One special instance of perceptual grouping is *contour integration* where local parts of an intersected contour are re-integrated into a continuous contour line, following the Gestalt rule of good continuation. Contour integration is typically investigated with a detection paradigm where subjects are presented with arrays of Gabor patches [2] (see Figure 1 for example stimuli). In some arrays, a subset of patches is aligned with a smooth invisible path so that they appear as local elements of a common contour. The task is to indicate whether or not the array contains a contour. Because the global form of the contour is unknown in advance, successful contour detection can only be achieved by correct grouping of the local elements.

It has long been assumed that contour integration emerges in a strictly bottom-up fashion. This view was put forward in psychophysical studies where contour detection performance was found to strongly depend on physical stimulus attributes. For example, an angle between adjacent elements of more than 30 degrees of orientation [3], or a distance of more than two degrees of visual angle [4] often renders the contour invisible. These results suggest that contour grouping is driven by orientation-selective neurons operating over a limited spatial scale. In order to explain how such a local mechanism can produce global perceptual wholes, Field et al. [2] proposed that activity within orientation-sensitive V1 neurons facilitates responses of neighboring neurons with a similar orientation preference while at the same time inhibiting neurons with a different orientation preference. This tendency results in a *local association field* of pair-wise linked contour elements that define the contour. In line with this idea, single-cell recordings from orientation-selective neurons in macaque V1 showed increased firing rates towards oriented stimuli if they were embedded within co-linearly oriented elements [5]. Increased activity in early visual cortex during contour detection was also found in human functional magnetic resonance imaging studies (fMRI; [6]–[7]). Together these results suggest that contour integration occurs without cognitive control, as a consequence of neural organization in primary visual cortex.

However, more recently this assumption was placed in question by the results of a perceptual learning study [8]. Similar to their former study [5], Li et al. [8] presented short oriented lines to the receptive fields of monkey V1 neurons and recorded their firing rates in situations where the line was presented in a context of co-linearly arranged (contour) or randomly oriented (non-contour) lines. They found that contextual modulations, as observed in [5], critically depended on the learning state. Untrained monkeys in a passive viewing task did not show differential neural responses to contours and non-contours. In contrast, the same monkeys showed clear contextual modulations after training and with an active detection task. In a third step, the authors showed that the previously observed contextual modulations in V1 disappeared if the trained monkeys were anesthetized. These results show that V1 responses to contours are mediated top-down from higher visual brain areas. More specifically, the results suggest that this control is administered by up- or down-regulating contextual modulations in orientation-sensitive V1 neurons. If the contour was not task-relevant, then no contextual modulation of V1 responses occurred. If the contour was relevant, then the spike rates of the recorded neurons increased monotonically with the length of the contour in which the target line was embedded. This suggests that contextual modulation was up-regulated such that contextual information was integrated over successive elements of the contour. A similar response enhancement in primary visual cortex due to attention has previously been found in a curve-tracing task [9]. Firing rates of V1 neurons were enhanced if their receptive fields were on a curve connecting a fixation dot and a target circle, but not if the curve was connected with a task-irrelevant distractor circle. The authors suggested that, if the trace is attended, horizontal connections in V1 propagate the firing rate modulations to neurons responding to neighboring segments of that contour. No propagation occurs if the contour is unattended so that no contextual modulation of V1 responses shows up.

Thus, on the one hand, contour integration is thought to arise in a bottom-up manner by facilitating V1 responses to line segments that are presented in a context of co-linearly oriented elements [2], [5]. On the other hand, it is known that such contextual modulations depend on task demands, suggesting that they can be regulated in a top-down fashion [8]. Little is known as yet about the neural mechanisms involved into this top-down control. In the present EEG study we aim to bridge this gap in our knowledge by investigating top-down control during contour grouping. Subjects were presented with pairs of contour and non-contour stimuli in a two-interval forced choice (2IFC) paradigm. The stimuli appeared in a randomized order, with the task to indicate whether the first stimulus ('S1') or the second stimulus ('S2') contained the contour. Two-interval forced choice (2IFC) tasks are well established in visual psychophysics. They are typically used to derive sensitivity measures by presenting noise stimuli together with stimuli containing a signal at variable intensities around the detection threshold [10]–[11]. According to the conventional ‘difference model’ of 2IFC performance [12], observers select a response by comparing the sensory evidence for a target being present in the first interval with that obtained in the second interval. Thus, observers judge the relative signal strength experienced in S1 and S2 displays rather than the absolute signal intensity.

We here use a 2IFC task to examine top-down control in contour grouping. Our assumption was that subjects performing a two-interval forced-choice task show top-down preparatory brain activity in the inter-stimulus interval between S1 and S2, depending on the sensory evidence for a target experienced during the presentation of S1. Similar to the strategy applied in psychophysical investigations, we mixed non-contour stimuli with near-threshold (response accuracy ∼ 75%) contour stimuli. If a high-signal strength (strong sensory evidence) was experienced during S1, then it was likely that S2 would not contain the target contour. Consequently, subjects should be less apt to group the contour elements presented in the following stimulus. In contrast, if a low signal intensity (weak sensory evidence) was experienced during the presentation of S1, then the target contour was likely to occur in S2. As a result, preparatory brain activity facilitating contour integration should show up prior to S2 presentation. The experienced signal intensity should be larger if contour stimuli are presented so that we expected less preparatory brain activity if S1 contained a contour compared to a situation if a non-contour was presented. However, it is important to note that the noise stimuli contained random co-linearities producing some sensory evidence for a contour presented in that interval. Thus, subjects could not rely on the sensory evidence for a target obtained during S1 alone.

We exploited two advantages of the EEG technique. First, EEG provides whole-brain measures of neural activity. This allows for investigating lower and higher brain areas at the same time, as opposed to previous studies [5], [8] where neural activity was exclusively recorded from V1 neurons. Secondly, compared to other whole-brain techniques, the EEG can be sampled with a high temporal resolution and so allows for the investigation of synchrony within brain oscillations. Oscillatory brain responses are thought to reflect rhythmic changes in neural excitability, where each cycle contains a time window where the sensitivity for synaptic input and spike output is maximal [13]. By synchronizing time windows of maximal excitability, distant neural populations can be transiently linked into neural assemblies that jointly process a given task. Brain oscillations are a means for investigating such multi-site neural communication.

Preparatory brain activity, as investigated in this study, often leads to a local power decrease within alpha band (8–12 Hz) oscillations prior to the presentation of a target stimulus. The power decrease is topographically specific and is thought to reflect excitation within task-relevant brain areas [14]–[16]. We expected posterior modulations in alpha power prior to the presentation of the S2, contingent on the type of stimulus (contour vs. non-contour) presented as S1. We were also interested in long-range synchrony between distant brain sites. This can be measured as the degree to which phase differences between oscillations recorded at two electrodes are constant in repetitive trials (phase-locking value, PLV; [17]). We expect to find increased long-range synchrony between higher and lower visual brain areas prior to the presentation of a contour in S2, in line with earlier studies [8].

## Methods

### Subjects

Twenty students of the University of Regensburg participated in the experiment. After an initial data screening, one subject was excluded from the further analysis due to excessive muscle artifacts in the EEG data. Another five subjects were excluded because their performance was at chance level as revealed by binomial tests. Thus, fourteen participants (six female, eight male, aged 19–32 years) remained in the sample. Based on self-report, all of them were right-handed, had no neurological disorders and normal or corrected-to-normal vision. The participants gave written informed consent prior to the experiment. Ethical approval was not required according to the institutional guidelines for ethics standards at the University of Regensburg, Institute for Psychology.

### Stimuli

In each trial, two stimuli were successively presented (Figure 1A and B). One stimulus contained a path of collinearly oriented Gabor elements that were embedded within an array of randomly oriented Gabors (‘contour stimulus’). The other stimulus contained randomly oriented Gabors only (‘non-contour stimulus’).

Gabor elements are oriented sine wave gratings that are multiplied with a two-dimensional Gaussian wave plane. Their luminance distribution G(x, y) can be described by the equation 1

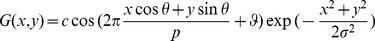
(1)where values *c*, *p*, θ and φ define properties of the sine wave grating. Value *c* is the Michelson luminance contrast, θ is the orientation, *p* is the wavelength, and φ is the phase of the grating. Sigma (σ) is the standard deviation of the Gaussian envelope. The wavelength of the carrier sine wave *p* was set to 8 pixels, resulting in a spatial frequency of 3 cycles per degree. The phase φ was zero so that the sine grating was even symmetric to the center of the Gaussian envelope. The Michelson contrast *c* was set to 0.9. The background luminance as well as the average luminance of a Gabor element was 4.3 candela per square meter. Sigma was set to 0.17 degree, which is approximately 1/2 of the wavelength *p*.

The stimulus area subtended a 14.3° square that was vertically and horizontally aligned to the screen center. It was subdivided into a 10 by 10 grid, resulting in 100 equally sized cells with an edge length of 1.4°. The strategy for generating contour displays was comparable to that described in [18]. In a first step, a trace of ten invisible line segments was constructed that served as a backbone for the Gabor path. The trace had a random starting point at the left or at the upper half of the stimulus area and then propagated through the stimulus display by successively adding line segments with a length of 1.73±0.33°, corresponding to the mean distance between adjacent Gabor elements in the cell grid (Figure 1). The curvature of the trace was controlled by the angle α between adjacent line segments, which was set to 23°. One Gabor element was placed at the center of each line segment, parallel to the line orientation Θ. The Gabor orientation was jittered by adding a random value drawn from a uniform distribution of ±10°, Δα. This led to slightly uneven contours that are relatively hard to detect. A hard task was desirable for our purpose because it would increase the need to attend to the stimuli, compared to a situation with highly salient contours that might pop out of the stimulus display.

Finally, cells that were not occupied by a path element were filled with randomly oriented Gabor elements. Their position was jittered by ±9 pixels (0.4°) relative to the cell center. In cases where adjacent Gabor elements would have overlapped, the cell was left empty. If the algorithm produced more than 8 empty cells, the whole contour stimulus was discarded and a new solution was computed. These safeguards assured us that the contours could not be detected by local pattern irregularities. Stimuli were also discarded if the contour traces propagated out of the stimulus area or if they formed circle segments.

Non-contour displays were obtained by a simple manipulation of the formerly constructed contour displays. Adjacent contour elements were rotated in opposite directions by 45 degrees, leading to a disruption of the contour impression. Furthermore, the distracter elements were rotated by a random value. Thus, the orientation of the single Gabor elements was different but their number and the positioning was identical in contour and non-contour stimuli (see Figure 1B).

### Procedure

Subjects were seated in a sound-attenuated, electrically isolated chamber (Industrial Acoustics GmbH) in front of a monitor with a viewing distance of 40 cm. A chin rest ensured that the distance remained constant and that the head position was centered at the screen. The stimuli were presented on a 17′′ flat screen monitor with a resolution of 1280×1024 pixels and a vertical refresh rate of 60 Hz. The power supply of the monitor was located outside of the recording chamber to reduce electromagnetic noise.

Trials started with a 1000 ms presentation of a black fixation cross on an otherwise uniformly gray screen. Then, one contour display and the corresponding distracter display were presented in a pseudo-random order for 200 ms each with a stimulus onset asynchrony of 1500 ms (for a depiction of a typical trial sequence see Figure 1A.). The long inter-stimulus interval guaranteed that no cues based on local apparent motion of the Gabor elements could aid contour detection [19]. The participant’s task was to indicate on each trial whether the contour was embedded within the first or within the second display by pressing the left or the right arrow key, respectively, on a conventional PC keyboard after the second stimulus was presented on each trial.

Subjects used their index finger and the middle finger of the same hand for responding. Half of the subjects responded with the right hand, and half of them responded with the left hand. After a button press, the blank screen remained for a short time interval before the next trial started. The time interval was normally distributed with mean 500 ms and standard deviation 25 ms. There was no feedback after single trials. However, subjects received feedback about their overall performance after each block of trials. The experiment consisted of 16 blocks with 30 trials each. The participants were explicitly informed that the presentation order – contour in the first interval or contour in the second interval – was randomized over all 480 trials. Thus, it was not possible to predict the presentation order for later occurring trials from those that were administered in earlier trials within one block.

### EEG Recording

EEG was recorded from 62 equidistant electrodes that were mounted in an elastic cap (EasyCap, Herrsching-Breitbrunn, Germany) and were referenced to FCz during recording. Impedances were kept below 10 kOhm. The signals were digitized at a rate of 500 Hz (BrainAmp MR plus, Gilching, Germany). Built-in analog hardware filters were used to limit the bandpass to 0.1–100 Hz. To control for eye-movement artifacts, the vertical electro-occulogram was recorded from an electrode placed below the left eye.

### Data Analysis: Behavioral Data

In order to investigate whether the subjects were biased to either attending to the first or to the second interval within one trial, reaction times and error rates were compared between those trials where the contour appeared in S1 or in S2. To control for outliers in reaction times, the fastest and the slowest 5% of responses per condition were eliminated. Trials with incorrect responses were also excluded. Statistics were computed with the free R language for statistical computing (R Development Core Team, 2008).

Because in the 2IFC task only one response is required for each pair of contour and non-contour stimuli, the stimulus type (contour or non-contour) was not a factor in the analysis of the behavioral data. Rather, the overall reaction times and error rates are given.

### Data Analysis: EEG Data

#### Preprocessing

The continuous EEG data were segmented into epochs from −2500 to 3500 ms, centered on the onset of the first stimulus within each trial. Epochs containing electrode or movement artifacts were removed. Also, trials with incorrect behavioral responses were discarded. The pre-cleaned data were subjected to an infomax independent components analysis [20]. Artifactual components related to eye blinks, eye movements, or tonic muscle activity were identified by visual inspection and removed from the data. Only clearly identifiable artifactual components were removed, leading to a moderate rejection rate of 7.41% ±4.55% of all components on average (mean ± standard deviation). The remaining components were back-projected into EEG signal space. Epochs were again inspected and rejected if they contained residual artifacts. Due to the overall large number of trials, stringent criteria could be adopted for trial rejection without leaving a too small amount of data for the analysis. On average, 236 trials (range 170–280) remained after trial rejection. The cleaned data were finally re-referenced to an average reference value.

The time-frequency decomposition was achieved by multiplication in the time domain. A sinusoid comprising 7 cycles of the target frequency was convolved with a time series (a data segment) of the same length, revealing a power estimate for the sample point representing the center of the data segment. This was done for successive data segments in steps of 10 ms, and for frequencies from 4 to 30 Hz in steps of 1 Hz. In a fixed-cycle approach as used in this study, the length of the time window for convolution decreases with increasing center frequency. The window length, in seconds, can be computed by multiplying the reciprocal value of a center frequency with the number of cycles included. For example, the length of the time window for a 4 Hz oscillation was 1/4×7 = 1.75s, and that for a 30 Hz oscillation was 1/30×7 = 0.23s. The corresponding frequency resolution, in Hz, is the reciprocal value of the window length in seconds. For example, the frequency resolution was 1/1.75 = 0.57 Hz for a 4 Hz oscillation and 1/0.23 = 4.3 Hz for a 30 Hz center frequency.

Before convolution the data segment was tapered with a Hanning window. By doing so, the data were weighted so that sample points near the event on which the time window is centered contribute stronger to the filtering output than sample points that are farther away from the event. In a time interval with n = 1…N samples, the weighting coefficients of a Hanning window are given by the function w(n) = 0.5×(1−cos((2×pi×n/N)). For example, in a data segment comprising 175 sample points, a signal occurring ±30 samples relative to the center of the segment is weighted by the factor 0.5×(1−cos((2×pi×(88−30)/175)) = 0.745. That is, assuming a 10 ms sampling interval, a signal occurring 300 ms prior to the event on which the time window was centered was attenuated by more than 24.5%.The latency after which a given attenuation is reached depends on the length of the data segment. For a 4 Hz oscillation, a 25% attenuation is achieved ±300 ms relative to the event on which the time window is centered, and for a 30 Hz oscillation it is achieved after ±40 ms.

A better resolution in time could be achieved by using shorter time windows for the time-frequency decomposition. However, shorter time windows produce a coarser frequency resolution. The 7-cycle window used for filtering 4 Hz oscillations was 1.75s long so that a frequency resolution of 0.57 Hz could be achieved. For comparison, if the data segment covered only one cycle (0.25s), a frequency resolution of only 1/0.25 = 4 Hz could be obtained. This is clearly insufficient considering that such a filter would not separate between oscillations in the delta (<4 Hz) and theta frequency band, or between theta and alpha frequencies. A fixed-cycle method as used in this study is a convenient way for dealing with the trade-off between frequency resolution and time resolution. The method reveals a fine frequency resolution at lower frequencies where it is needed to separate between delta, theta and alpha band oscillations. At the same time, it reveals a good temporal resolution at higher frequencies where oscillations occur within broader (beta and gamma) bands so that a fine-grained frequency resolution is less important. The fixed-cycle method has been successfully used in a wide range of EEG studies [21]–[22]. A 7-cycle window is an especially reasonable choice when investigating top-down control where effects are expected to occur in lower (theta and alpha) frequency bands [23].

In order to investigate event-related changes in the oscillatory brain activity, it is important to use a baseline covering a time period shortly before stimulus onset. Changes in brain activity relative to this baseline can then be directly interpreted as a result of the stimulation. To examine event-related power changes, the percentage power increase or decrease relative to a baseline period was computed as 100 × [A(t,f) – Ab(f)]/Ab(f), where A(t,f) is the amplitude at time t and frequency f, and Ab(f) is the mean amplitude over the baseline interval. A normalization to baseline activity is necessary due to large inter-individual differences and inter-trial variability in oscillatory brain activity. The baseline was set from −600 to −100 ms, relative to stimulus onset. It ended 100 ms before stimulus onset in order to minimize possible contributions of post-stimulus oscillatory brain activity to the baseline estimate. To reveal a measure representing all trials of a condition, the obtained single-trial event-related power changes were averaged over all trials belonging to one condition (contour or non-contour, S1 or S2). Only trials with correct behavioral responses were considered. EEG post-processing was accomplished with custom routines and the Fieldtrip toolbox [24] for MATLAB environment (The Mathworks, Inc.).

The phase-locking value (PLV) was computed with the same filter and baseline settings as described above. The phase information is retained in the corresponding Fourier spectra of the frequency decomposition. It can be illustrated as a vector within unity circle that represents the phase angle φ of an oscillation at a given time point *t* and at frequency *f*, φ (*t, f*). The PLV measure quantifies the consistency of phase differences between signals obtained at two different sensors [17]. It is computed as φ_1_(*t, f*)–φ_2_(*t, f*) for each trial *n* that belongs to one condition and then averaged over trials by taking the circular mean. The length of the resultant vector is PLV. A value of 1 indicates constant phase differences, and a value of 0 indicates that they are random. The PLV between two sites can be artificially high due to volume condition where the sensors pick up activity of the same neural source. In order to reduce the impact of volume conduction, sensor data were transformed to current source density prior to PLV calculation using the CSD toolbox for MATLAB [25]. Furthermore, because measures of phase consistency are affected by trial count [26], the number of trials per condition was adjusted prior to PLV calculation by drawing a random sample of trials from the condition with the larger trial count. This was done separately for each subject. On average, 8% of trials had to be discarded in order to achieve an equal trial count between conditions. Note that a similar trial selection was not performed for the power analysis. The mean of the single trial power values is an unbiased estimator of the average power within the condition, i.e., it is not affected by trial count. For this analysis it is advisable to keep the maximum number of trials available, in order to achieve a high signal-to-noise ratio.

#### Statistical analysis

Power differences between contour and non-contour conditions were examined with paired t-tests at each frequency and at each time point of the EEG segment. This was done at all 62 electrodes simultaneously.

To correct for multiple comparisons across electrodes, a nonparametric randomization procedure was used. The procedure is described in detail in [27] and has been successfully applied in previous MEG and EEG studies [28]–[29]. It identifies clusters of spatially contiguous electrodes where the results of the t-tests were significant (with p<.05, two-sided). The t-values of all electrodes belonging to one cluster are summed up to reveal a cluster level statistic. Then, random permutations of the data are drawn by exchanging the data between experimental conditions within the participants (1000 permutations in the present study). The maximum cluster level statistic is recorded after each permutation run, revealing a reference distribution of cluster level statistics. The randomization is done separately for significant electrodes showing positive or negative t-values. Thus, it reveals separate reference distributions for positive (contour>non-contour stimuli) and for negative differences (non-contour>contour stimuli). Cluster-level p values can then be estimated as the proportion of the values in the corresponding reference distribution exceeding the cluster statistic obtained in the actual data. This procedure effectively controls for multiple comparisons over electrodes.

Significant time and frequency ranges for the data presentation were selected from the results of the cluster permutation procedure. To further reduce the probability of false positive tests, results were only considered if they occurred within two or more contiguous frequency bins (2 Hz) and within six or more consecutive time bins (60 ms). A similar strategy has already been used in an earlier publication of our group [30]. In order to obtain head topographies, the power was averaged within significant time and frequency ranges, separately for contour and non-contour stimuli, and subjected to dependent t-tests for each electrode. The head topographies show the resulting t-values. Additionally, head topographies on the corresponding power differences are provided. Individual head topographies for a given effect were transformed to current source density to reduce the effect of volume conduction. Following the procedure described in [31], the individual head topographies were z-transformed and then averaged over subjects to reveal a normalized grand mean average of the current source density maps (zCSD).

PLV measures in the contour and non-contour condition were compared by means of paired t-tests at each time point and frequency of interest, and for all possible channel combinations. For our 62 channel net, the number of possible channel pairings was (62×62−62)/2 = 1891. The number of electrode pairings with significant PLV differences was recorded for each time and frequency bin. To determine the significance of PLV increases/decreases, 1000 random permutations of the data were drawn at random time points within each frequency, and the number of significant electrode pairings was recorded after each run. This was done separately for positive differences (contour>non-contour) and negative differences (non-contour<contour). The p value for PLV increases or decreases was then computed as the proportion of runs where the permutation procedure revealed a larger number of significant pairings than obtained in the actual data.

Complementary to the primary analysis of power and PLV differences between contour and non-contour S1 and S2 displays, two additional analyses were performed. Firstly, in order to investigate the specificity of post-stimulus differences for either S1 or S2 displays, the obtained effects were compared by means of a two-way analysis of variance (ANOVA) for repeated measures including the factors CONTOUR (contour, non-contour) and DISPLAY (S1, S2). The details on this analysis depend on the results of the primary analysis and are provided in the results section ‘Comparisons of post-stimulus activity in S1 and S2 displays’. Secondly, it was examined whether possible differences in pre-stimulus power or PLV between contour and non-contour stimuli in S2 were due to an *in*crease of brain activity or due to a *de*crease of brain activity in either condition. To this end the brain activity in the contour and non-contour conditions was compared with the average brain activity during a resting period in the interval-trial interval by means of paired t-tests. An increase in brain activity, relative to resting levels, would indicate an active preparation for processing the upcoming S2 display. Alternatively, a decrease in brain activity would indicate that subjects ignored the upcoming S2 display. Details on this analysis are provided in the results section ‘Comparison of pre-stimulus activity in S2 displays with resting activity’.

## Results

### Behavioral Data

Subjects responded correct in 71.01±4.8% (mean ± standard deviation) of trials on average. This was significantly different from chance performance, t(13) = 15.33, p<.001. The mean reaction time for trials with correct responses was 791±237 ms. The analyses revealed no performance differences between trials where contours were presented within S1 and S2 [S1: 801±216 ms, 70.95±6.92% correct; S2: 781±237 ms, 71.07±5.84% correct; both F(1,13) <2.4, p>.15]. Two of the subjects had a bias towards judging that the contour was in S1, and three subjects had a bias towards judging that it was in S2 [all χ2 (df = 1, n = 420) >5.67, all p<.05]. The number of subjects showing a bias towards responding ‘S1’ or ‘S2’ was not significantly above chance in either case (both p>.9 as revealed by binomial test).

### EEG Data

Differences in oscillatory brain activity associated with contour and non-contour stimulus displays are presented in different sections for S1 and S2. The data are shown from −0.6 s to 0.6 s relative to S1 and S2 onsets, respectively. This time selection is derived from previous EEG on preparatory brain activity as well as post-stimulus activity associated with the presentation of contour stimuli [14], [32]–[34]. Note that the same pre-trial baseline was used for both S1 and S2 stimuli.

#### S1 displays

**Figure 2:**
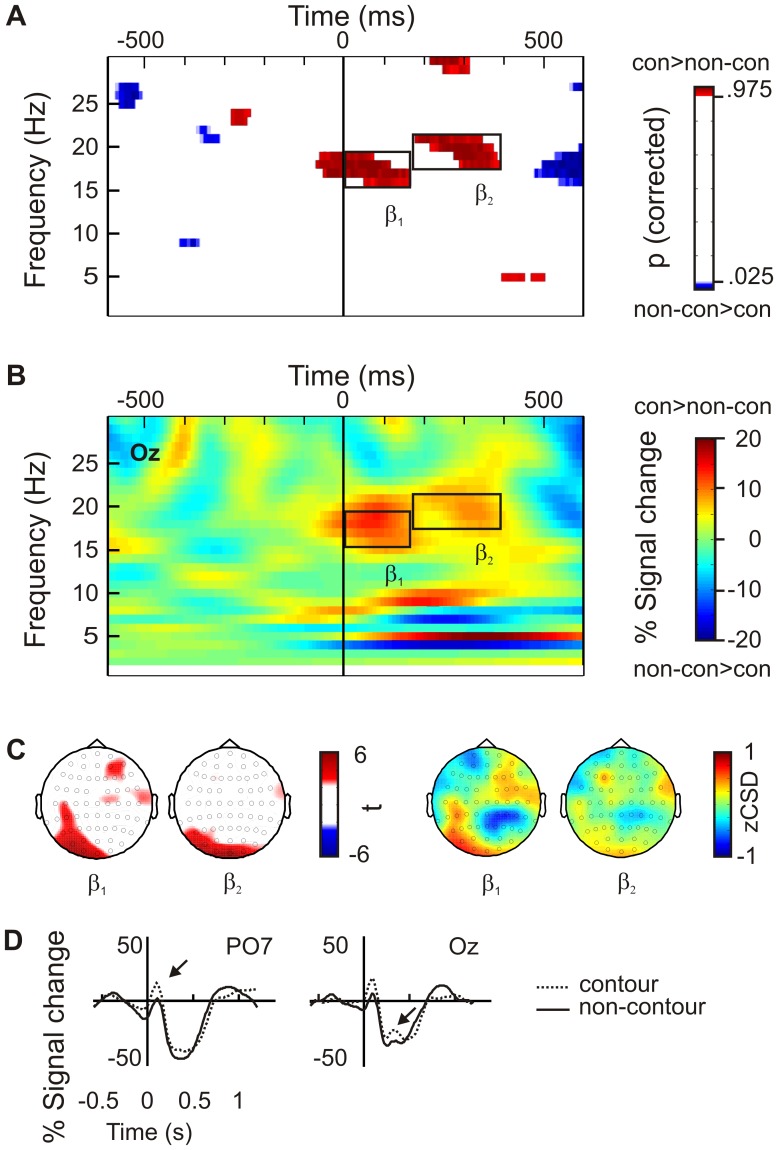
This figure shows the results of the power analysis for stimuli presented in the first interval within a trial (S1). **A** Time-frequency plot showing the results of cluster permutation statistics (p-values) on power differences between contour and non-contour stimuli. Red color indicates a significantly higher power and blue color indicates a significantly lower power for contour stimuli. P-values are two-sided and corrected for multiple comparisons across electrodes. There are two obvious effects, marked by black frames, within lower and higher β frequencies (ß_1_, ß_2_). **B** Shows the corresponding power differences, contour minus non-contour condition, at representative electrode Oz. **C** Head topographies on mean power differences between contour and non-contour stimuli (t-values), and z-transformed current source density maps on the corresponding power differences (zCSD). The power and zCSD values were averaged within significant time and frequency ranges as indicated in sub-figure A (ß_1_, ß_2_). Non-significant t-values are masked. Both effects have a parieto-occipital focus. **D** Waveforms showing mean power changes for contour and non-contour stimuli at selected electrodes (ß_1_: PO7, ß_2_: Oz). Black arrows point to the time points of peak differences.

The results of the cluster permutation test on power values are summarized in Figure 2A. Red color indicates significantly increased activity (two sided p, upper tail) and blue color indicates significantly decreased activity (two sided p, lower tail) in the contour compared to the non-contour condition. Additionally, the corresponding power differences are shown for one representative electrode (Oz, Figure 2B). Please note that the applied cluster permutation procedure corrects for multiple comparisons across electrodes, but not for multiple comparisons across time and frequency bins. Thus, spurious differences between contour and non-contour trials occurred in the baseline period (Figure 2A). They were actually not significant according to the criteria defined in the method section (9 Hz), or occurred in frequency bands irrelevant for the main analysis (>21 Hz). Thus, the multiple correction procedure for the main analysis worked as intended.

Two prominent effects can be identified. Firstly, contour compared to non-contour stimuli induced a higher power in lower beta frequencies (β_1_ in Figure 2A and 2B: 15–19 Hz) from 0 to 150 ms relative to stimulus onset. Figure 2C shows the head topographies for this effect (t-map and zCSD). As can be seen in the t-map, the β_1_ difference had a left parieto-occipital topography covering nine electrodes (P3, O1, P7, Oz, CP5, PO3, C5, P5, and PO7; all t >2.23, all p<.05). This is also confirmed by the head topography showing the grand mean average zCSD. The largest t-statistic occurred at electrode PO7, t(13) = 4.83, p<.001. For this electrode, waveforms are provided for contour and non-contour conditions representing the power change relative to baseline activity (Figure 2D, left). Beta activity increased shortly after stimulus onset, where the increase was larger for contour compared to non-contour stimuli. The difference had a maximum 80 ms after stimulus onset. An inspection of Figure 2A indicates that small differences in the β band were already present prior to the actual stimulus onset. This difference was likely produced by the strong β_1_ effect that might have extended into the baseline period due to the limited temporal resolution of the time-frequency decomposition. Alternatively, it is possible that the β_1_ effect actually emerged prior to the stimulus presentation and continued into the post-stimulus interval. In order to rule out that possibility, we repeated the analysis on the head topography with an extended baseline covering the whole pre-stimulus interval up to the stimulus onset (−600 to 0 ms). If the difference in beta power was already present prior to stimulus presentation, then any post-stimulus differences should diminish in this analysis. However, the pattern of results was the same as in the previous analysis, with only electrode P3 showing a positive trend instead of a significant difference [t(13) = 2.05, p = .06]. As a further control analysis, the mean difference in β_1_ activity between contour and non-contour stimuli prior to S1 onset was compared with the same difference after S1 onset. To that end, the β_1_ activity was averaged over a −100 to 0 ms period (where the peak pre-stimulus deflection occurred) and over a 0 to 100 ms period (where the peak post-stimulus deflection occurred) relative to S1 onset, respectively. The difference between contour and non-contour stimuli was larger in the post-stimulus interval compared to the pre-stimulus interval [9.33% vs. 6.12%, respectively; t(13) = −2.29, p<.05]. Thus, the post-stimulus differences between contour and non-contour stimuli cannot be explained by pre-existing differences in the baseline interval.

A second effect was seen in the higher beta band (170–380 ms, 18–21 Hz, β_2_ in Figure 2A and 2B). Again, there was increased beta activity for contour compared to non-contour stimuli. The β_2_ effect had a more bilateral and occipital topography covering seven electrodes (P3, O1, O2, P7, Oz, PO3, PO7; all t >2.28, all p<.05; see Figure 2C, t-map and zCSD). The t-statistic was maximal at electrode Oz, t(13) = 5.63, p<.001. The corresponding waveforms indicate that after an initial increase the power decreased in both contour and non-contour conditions (Figure 2D, right). The decrease was followed by a small positive peak, which was larger for the contour compared to the non-contour stimuli. The difference was maximal 320 ms after stimulus onset.

**Figure 3:**
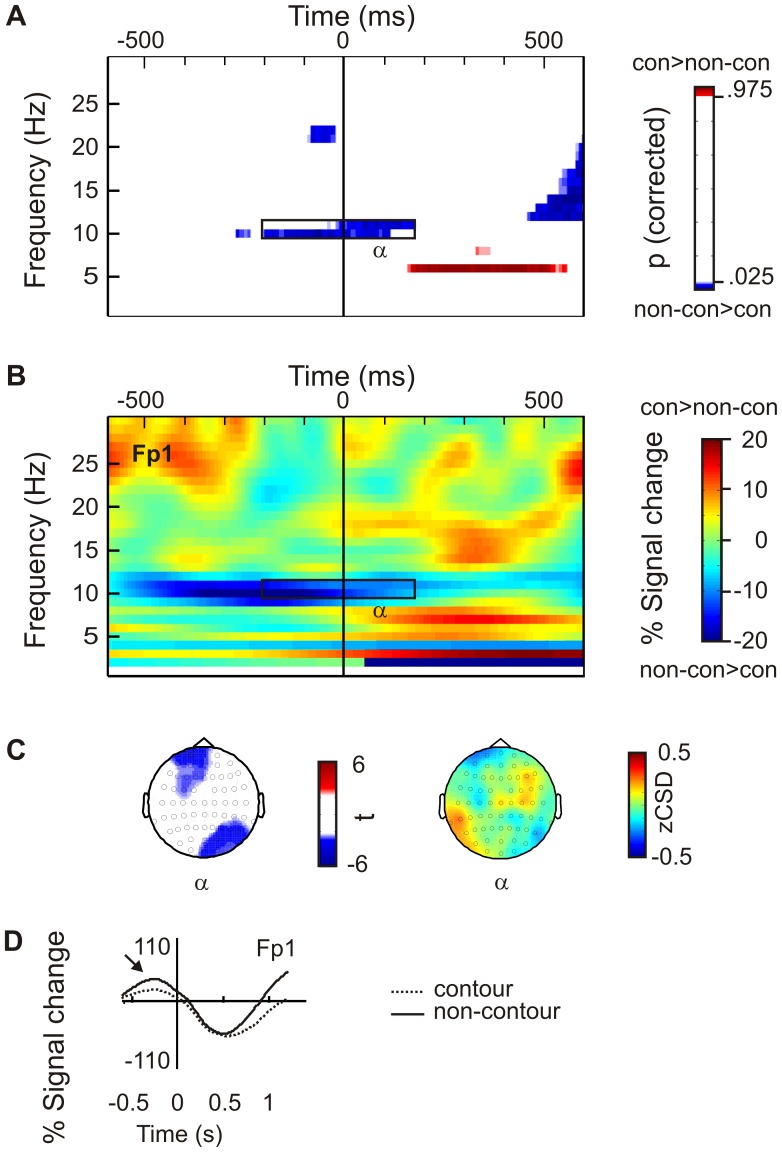
Same as Figure 2, but for stimuli presented in the second interval within a trial (S2). **A** The data show lower pre-stimulus alpha (α) band activity for contour compared to non-contour stimuli. **B** Power differences, contour minus non-contour condition, at electrode FPz. **C** Head topography reveal a left frontal and a right parieto-occipital focus for alpha power differences (t-map and zCSD-map). **D** Waveforms at Fp1 show a pre-stimulus increase in alpha power, which was larger for non-contour compared to contour stimuli.

**Figure 4:**
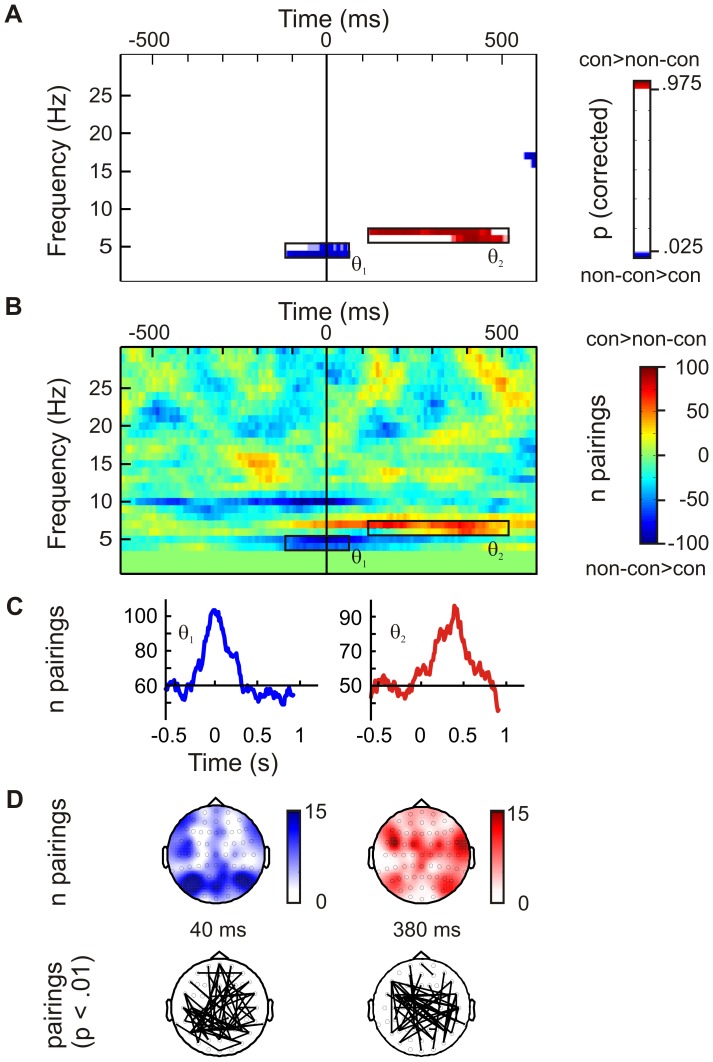
This figure shows the results of the PLV analysis for second-interval stimuli (S2). No PLV differences between contours and non-contours were found for first-interval (S1) stimuli. **A** Plot showing time and frequency ranges of PLV differences as obtained by randomization tests (p-values). The p-values refer to the number of electrode pairings where PLV was significantly increased (red) or decreased (blue) for contour compared to non-contour stimuli. Prominent effects were found within lower and upper theta frequencies as indicated by black rectangles (θ_1_, θ_2_). **B** Shows the number of electrode pairings where PLV was larger in the contour compared to the con-contour condition minus the number of electrode pairings where PLV was larger in the non-contour compared to the contour condition. **C** Waveforms showing the number of electrode pairings with significant PLV differences in the contour and non-contour conditions, as a function of time. A large number of couples exhibited a pre-stimulus PLV decrease (θ_1_, left) and a post-stimulus PLV increase (θ_2_, right) for contour- compared to non-contour stimuli. **D** Head topographies showing the number of pairings with significant PLV increases or decreases that a given electrode enters into (upper row). A subset of these couplings, where p<.01 for PLV differences, are illustrated by lines connecting electrodes (lower row). The θ_1_ effect (left) involved mainly frontal and parieto-occipital electrodes, whereas the θ_2_ effect (right) was found at frontal and temporal electrodes.

Figure 2A also indicates that there was an effect >500 ms after stimulus presentation. Such late effects were observed for both power and PLV measures and in S1 as well as in S2 displays (compare Figures 2A, 3A, 4A). They were generally long lasting (∼ 500 ms), covered a broad spectrum of alpha and β frequencies (∼ 10–20 Hz), and always indicated lower power or PLV for contour compared to non-contour stimuli. Since the same effect occurred in S1 and S2 displays and since it emerged only after 500 ms, it is safe to conclude that this difference is not related to top-down preparation for contour stimuli. This effect will thus not be further considered in this study.

With respect to PLV, no further differences between contour and non-contour stimuli were observed in S1.

#### S2 displays

The results of the power analysis for S2 contour and non-contour displays are presented in Figure 3A and 3B. The data showed a broad difference in the alpha frequency band (10–11 Hz, α in Figure 3A and 3B), with higher amplitudes in the non-contour compared to the contour condition. The effect began 280 ms prior to stimulus onset, lasting until 160 ms post-stimulus and had a left frontal [Fp1, F3, F5, Fz, FC1, F1, AF3, FC3, AF7, AFz; all t(13)<−2.6, p<.05] and right parietal topography [P4, O2, P8, Oz, PO4, P6, PO8, POz; all t(13)<−2.3, p<.05; see Figure 3C, t-map and zCSD]. The maximal t-value was observed at electrode Fp1, t(13) = 3.89, p<.001. The waveforms at this electrode showed a general pre-stimulus increase in α power that was larger prior to the presentation of a non-contour (i.e., on trials where a contour was presented as S1) compared to the presentation of a contour (i.e., on trials where a non-contour was presented as S1). The difference peaked 250 ms prior to the S2 presentation, 1450 ms after S1 onset (Figure 3D).

With respect to PLV, the data showed two differences between contour and non-contour S2 displays, both occurring in the theta frequency range. Figure 4A shows the results of the permutation statistics. Additionally, Figure 4B shows the number of electrode pairings where PLV was larger in the contour compared to the non-contour condition minus the number of electrode pairings where PLV was larger in the non-contour compared to the contour condition. Positive values (red color) thus indicate an increased phase-locking in the contour compared to the non-contour condition, and negative values (blue color) indicate the reversed effect. Most interesting was a decrease in lower theta PLV (4–5 Hz, θ_1_ in Figure 4A and 4B) for contour compared to non-contour stimuli, prior to the presentation of the stimulus. This significant difference occurred −120 to 50 ms relative to S2 onset. Figure 4C (left) shows the number of electrode pairings where PLV was significantly lower for contour compared to non-contour S2, as a function of time. One can see that the number of pairings increased up to the time of target presentation and decreased afterwards. The maximum was reached between −40 and 0 ms where 104 electrode pairings were affected. Figure 4D (left) shows a head topography where the color codes represent the number of pairings with significantly reduced PLV for each electrode involved. Furthermore, a head topography is shown where electrode pairs with a significant PLV decrease are connected with lines. To make the effect visible, these lines are depicted only for a subset of pairings with p<.01. As this figure shows, the θ_1_ effect mainly involved frontal and parieto-occipital electrodes.

A subsequent increase in higher theta PLV (6–7 Hz, 110–510 ms; θ_2_ in Figure 4A and 4B) was evident for contour compared to non-contour stimuli. The effect peaked 380 ms after S2 onset where 97 electrode pairings with significantly increased PLV were observed (Figure 4C, right). As compared to θ_1_, this effect involved more fronto-central and temporal electrodes (Figure 4D, right).

#### Comparison of post-stimulus activity in S1 and S2 displays

Post-stimulus activity (β power and θ_2_ PLV) was compared between S1 and S2 displays in order to evaluate the specificity of the effects. For each subject, the mean power at significant electrodes obtained within the β_1_ and β_2_ time and frequency range were averaged and subjected to a two-way analysis of variance (ANOVA) for repeated measures with the factors CONTOUR (contour, non-contour) and DISPLAY (S1, S2). The interaction between both factors was significant, indicating that the β power increase was specific for S1 displays [F(1,13) = 14.94, p<.01]. The β power was larger for contour compared to non-contour stimuli presented in S1 displays (−12.57% versus −19.64% signal change; compare Figure 2D). This difference was not seen for S2 displays (−23.2% and −19.64%, respectively). Note that the β power difference for S1 displays emerged from an initial power increase relative to baseline levels for contour stimuli. Beta power then decreased for both contour and non-contour conditions so that the average power change within the investigated time range is negative.

An analogous analysis was performed on the mean θ_2_ PLV in the 110 to 510 ms post-S2 time range. Compared to S1 displays, the θ_2_ PLV increased in trials where a contour stimulus was shown in S2 (S1: 0.02, S2: 0.07), and it decreased if a non-contour stimulus was shown (S1: 0.08, S2: 0.02). Consequently, the data showed an interaction between the factors CONTOUR and DISPLAY, F(1,13) = 151.5, p<.001.

#### Comparison of pre-stimulus activity in S2 displays with resting activity

Finally, we investigated whether the pre-stimulus α power and θ_1_ PLV differences between contour and non-contour stimuli observed in S2 displays were due to an increase or decrease of activity relative to baseline levels. Increased brain activity would indicate an active preparation for processing the upcoming S2 display, whereas decreased brain activity would indicate that subjects ignored the upcoming S2 display in this condition.

**Figure 5:**
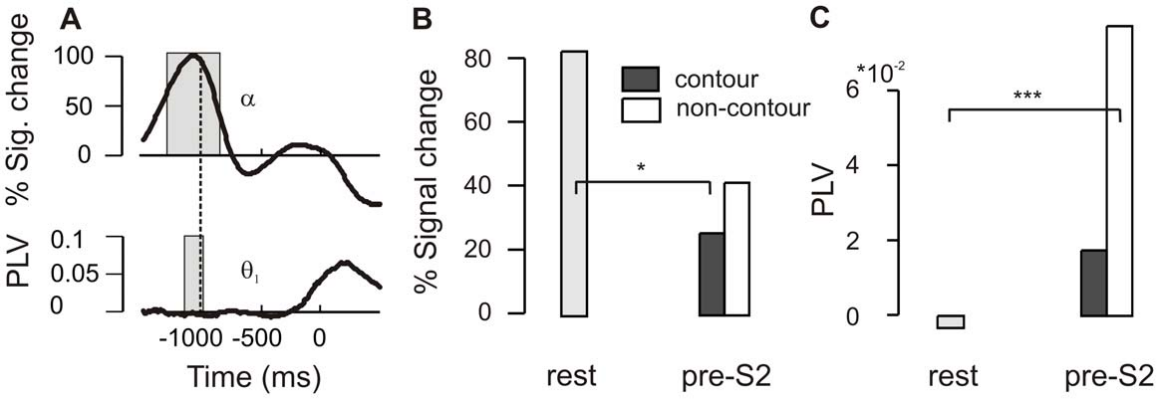
This figure shows the results of a comparison of pre-stimulus activity in S2 displays with resting activity. A Grand mean average waveforms showing α power and θ_1_ PLV prior to S1 onset. The dashed vertical line marks the onset of the fixation cross. Values within the time ranges indicated by the grey rectangles were averaged to obtain resting levels of α power and θ_1_ PLV, respectively. **B** Bar graphs showing differences in pre-stimulus α activity between contour (black bars) and non-contour (white bars) S2 displays (−280 to 160 ms) and the average α power (grey bars) in a resting period of the same length (−1300 to −860 ms). **C** Mean pre-stimulus θ_1_ PLV obtained for contour and non-contour S2 displays (−120 to 50 ms), and the average θ_1_ PLV in a resting period of the same length (−1160 to −990 ms). * p<.05, ** p<.01, *** p<.001.

For investigating the α power effect, the mean power in the contour and non-contour S2 displays during the time period where α power differed was compared with the mean α power in a resting situation during the inter-trial interval. The −600 to −100 ms baseline used in the primary analysis is not an appropriate reference for this analysis because activity in the relevant brain areas might already be increased due to the temporal expectation of S1 [35]. Therefore, a new reference period was used covering the time between two successive trials where the subjects were at rest. In order to identify a suitable time range, the time course of the grand mean alpha power was visually inspected and a segment of the same length as the observed α power effect (440 ms) was chosen from the waveform. The highest alpha power, indicating the lowest excitation, occurred −1300 to −860 ms relative to the onset of S1 (Figure 5A, upper row). This time period, referred to as the resting interval in the following, was used for the analysis. The brain activity was averaged over contour and non-contour trials in the resting interval because a distinction between the conditions is not meaningful at this time point. To make clear that the analysis is on pre-stimulus activity relative to S2 onset, we will denote the relevant time periods as pre-S2 in the following.

The results are depicted in Figure 5B. The pre-S2 α power for contour stimuli was significantly reduced compared to resting levels of α, t(13) = 2.31, p<.05. No such difference was observed for non-contour stimuli, t(13) = 1.74, p>.1.

A similar analysis was conducted on the pre-stimulus θ_1_ PLV data. The mean θ_1_ PLV for contour versus non-contour stimuli obtained at significant electrodes during a −120 and 50 ms in S2 displays was contrasted with the mean PLV obtained in the in a resting period of the same length (170 ms). As for the α power analysis, a suitable time range that could serve as a reference for resting activity was obtained by visual inspection of the grand mean θ_1_ PLV waveform. Low PLV values indicating a state of low connectivity occurred in the inter-trial interval, −1160 to −990 ms relative to the onset of S1 (Figure 5A, lower row). The result can be seen in Figure 5C. Pre-S2 θ_1_ PLV in non-contour trials was significantly increased relative to resting levels, t(13) = −6.02, p<.001. No corresponding increase was found for contour trials, t(13) = −1.99, p>. 05.

## Discussion

We investigated the brain mechanisms of bottom-up and top-down processing in perceptual grouping. A single contour and a non-contour stimulus were presented in a random order in a two-interval forced-choice task where participants had to decide whether the first stimulus or the second stimulus contained the contour. Because the presentation order was randomized, S1 processing had to rely on bottom-up information. In contrast, S2 processing could be modulated contingent on the result of S1 processing (i.e., contour detected or not detected in S1).

The data showed clear differences in oscillatory brain activity associated with contour and non-contour stimuli. Moreover, and more importantly, brain responses were different in situations with and without prior knowledge about the content of the upcoming stimulus. Whereas contour compared to non-contour S1 produced larger post-stimulus β power, contour compared to non-contour S2 displays were associated with a lower pre-stimulus α power and a lower pre-stimulus θ PLV. A post-stimulus β difference, as observed for S1 contours, was not evident for S2 stimuli. The fact that brain activity evoked by contour stimuli was different in situations with and without prior knowledge is in line with the idea that top-down control is involved in contour grouping [8]. In addition to this previous study, our results suggest that this control is deployed within local α and long-range θ networks.

For S1 displays we found increased posterior activity in the contour compared to non-contour condition in lower beta (β_1_, 0–150 ms, 15–19 Hz) as well as higher beta frequencies (β_2_, 170–380 ms, 18–21 Hz). The timing and the topography of the effect are in good accordance with previous EEG and magnetoencephalography (MEG) studies that used contour detection tasks. Tanskanen et al [34] found a peak difference in evoked magnetic fields between contours and non-contours 215 ms after stimulus onset at parieto-occipital sensors. Likewise, Mathes et al. [33], [36] found a posterior difference 150 to 250 ms after stimulus presentation. Our β_1_ and β_2_ differences are similar to the previous findings with respect to the latency and the electrodes involved into the effect. The similarity of these results suggests that our paradigm worked as intended, in that the S1 condition was comparable to a conventional contour detection task with a single presentation interval.

The results for S1 displays emphasize the role of β oscillations for contour grouping. Two previous studies also suggest that there is a link between β oscillations and perceptual grouping [37]–[38]. Romei et al [38] applied rhythmic transcranial magnet stimulation (TMS) in either θ or β frequencies to right parietal cortex and asked their subjects to detect letters on either the global or local level of a compound Navon-type stimulus [39]. While θ frequency TMS improved performance for the global level task, β frequency TMS specifically benefited the performance for the local level. Accordingly, the increased β power in our study might indicate a more local mode of processing. Since similarities of local orientations are the relevant grouping cue in our study, a process by which the saliency of the local information is enhanced might facilitate contour detection.

A post-stimulus effect was also found for S2 displays where a significant number of pairings showed an increased θ_2_ PLV for contour compared to non-contour stimuli. This effect might be related to the behavioral response that we required after S2. Long-range θ synchrony has been found to occur at decisions points where subjects choose to select one action over the other ([40]–[41]; for a review see [42]). One sub-component of decision-making is the retrieval of choice-relevant sensory evidence that was obtained during stimulus presentation. It is conceivable that the increase in post-S2 θ PLV for contour displays is related to such evidence retrieval [43]. In a two-interval forced-choice task, subjects are required to compare the signal strength obtained during S1 presentation with that obtained during the presentation of S2 [12]. Irrespective of whether S1 or S2 contained the target stimulus, a decision on the correct response is made only after S2 presentation. For arrays presented in S1, no response selection is necessary. Thus, no θ PLV effect occurs in this situation. The θ PLV modulation after S2 onset might reflect a process of evidence retrieval for the required response selection. Since θ PLV was larger for contour compared to non-contour stimuli, the data also indicates that evidence retrieval is facilitated if the target stimulus is presented as S2.

Most interesting for the purpose of this study, the data revealed preparatory brain activity towards contour arrays shown in S2. Corresponding effects occurred, at the same time, within α power as well as θ_1_ PLV measures.

The α power effect occurred mainly over visual areas at occipital and parietal electrodes. Alpha amplitudes are considered to reflect inhibition within the affected brain regions [44]–[45]. Correspondingly, reduced α amplitudes indicate a higher neural excitability. Pre-stimulus modulations of α activity have been found for a number of different tasks [16], [46]–[47]. For example, spatial attention to the left side of space was found to produce an α power decrease in right visual cortex, and attention to the right side of space produced a left-hemispheric decrease in α power [14]. We here find a similar effect in a task where neither the spatial location nor a specific feature of the target object was predictable.

For the further discussion of the α difference between contour and non-contour stimuli in S2 displays it is an important question whether it emerged due to a *de*crease of α activity in the contour condition or due to an *in*crease of α in the non-contour condition. An α power decrease for contour stimuli would indicate an active preparation for contour processing in the S2 stimulus. Alternatively, an α power increase for non-contour stimuli would indicate that subjects ignored the S2 displays if a contour was presented in the first interval. Overall, our data support the former interpretation. Using resting levels of α as the baseline condition, we see significant power decrease for contours presented in S2 displays. A corresponding decrease was not observed for non-contour stimuli. Thus, the data support the interpretation that the α power differences between contour and non-contour stimuli presented in S2 displays were due to an active preparation for contour processing.

Notably, this result is different from those of earlier studies on preparatory brain activity. Remember that contour grouping is thought to emerge in a bottom-up fashion from local interactions between orientation-sensitive neurons. It is the relative orientation and spacing of local Gabor elements that define the target object. The absolute orientation of the Gabor elements was not predictive of the occurrence of a contour so that subjects could not focus on a specific target orientation. Furthermore, the global shape of the contour was random so that subjects could not pre-activate a corresponding object representation. A possible explanation on how an increase of neural excitability can aid target detection in our contour-grouping task can be derived from the local association fields approach [2]. Within this framework, a higher excitability of visual cortex should lead to a larger co-activation between neighboring neurons that are stimulated by contour elements. A similar feedback does not exist between non-neighboring neurons or between neurons with different orientation preferences. An increase of visual cortex excitability should therefore affect the activity of neurons that are part of a positive feedback-network to a larger extent than the activity of neurons outside that network. Consequently, the processing should be biased towards Gabor elements that are part of a global contour.

Modulations of α power prior to or during visual processing have been observed in a large variety of tasks [44]. It can be considered as a general brain mechanism of goal-directed selection that is not specific for the stimuli at hand. Pre-stimulus modulations of θ PLV are not very prominent, so the role of θ_1_ PLV for contour processing is less obvious. For an adequate interpretation of the PLV difference, it is again important to know whether the difference in θ_1_ PLV in S2 displays was due to a PLV decrease of in the contour condition or due to an increase in the non-contour condition. The data showed a significant increase in PLV from resting levels to non-contour S2 displays. Thus, as for the α power, the data showed an increased activity relative to a resting condition. This suggests that the modulation of θ_1_ PLV prior to S2 displays reflects an active preparation for upcoming stimulus processing.

Given that the observed pre-stimulus α power and θ_1_ differences reflect top-down preparation for contour processing, the question arises whether such preparation has positive consequences for behavioral performance. Previous studies on pre-stimulus α activity indeed often revealed that lower levels of pre-stimulus α power are associated with an enhanced performance in the associated task [14], [16]. For the present study one could therefore expect a correlation between performance and pre-stimulus (S2) α power or θ_1_ PLV differences in contour and non-contour conditions. We tested this prediction by subtracting, for each subject separately, the pre-S2 levels of α power and θ_1_ PLV in the non-contour condition from that found in the contour condition and correlating that difference with the individual error rates. The result was neither significant for α power (r = .08, p>.7) nor for θ_1_ PLV (r = −.39, p>.16), thus appearing to contradict the claim that they reflect top-down control in contour grouping. However, a 2IFC paradigm is not optimally suited for investigating correlations between pre-stimulus activity and performance. This is because the behavioral response does not only depend on the sensory evidence for a contour being present in S2, but also on the sensory evidence that was obtained during S1 presentation. For example, even if participants prepared themselves for contour processing and obtained some sensory evidence for a target in S2, they might still give an ‘S1’ response if there was also evidence for a contour in the first interval. Likewise, poor preparation and low sensory evidence for a contour in S2 might still lead to the correct response if the evidence exceeds that obtained during S1 presentation. A tight association between brain activity prior to S2 displays and behavioral performance cannot be expected in this type of paradigm. Consequently, the missing correlation with performance does not contradict the idea that pre-stimulus α power and θ_1_ PLV reflect top-down preparation for contour processing.

As previously mentioned, long-range θ synchronization is thought to occur during the integration of task-relevant information existing in specialized but distributed brain areas [42]. According to this view, θ synchronization orchestrates the use of information that persists in the system at a given point in time. However, this interpretation does not fully comply with our results. The accumulation and selective use of sensory evidence become relevant only after a sensory event has occurred. In contrast, the θ_1_ decrease for upcoming contour arrays observed in our data peaked exactly at S2 onset. This suggests that the effect is not related to post-sensory selective retrieval of information, but rather to the sensory processing of the stimulus itself. Such an early modulation would also be more in line with the results of Li et al.’s [8] monkey study. The authors demonstrated an effect of top-down control on contour grouping at the level of V1 neurons, showing that control occurs before the sensory evidence enters a stage of response selection. Finally, we must consider the fact that the post-stimulus brain responses differed towards contour arrays in S1 and S2. It is hard to see how top-down selection of sensory information could produce this result. On the other hand, it is possible to explain the difference by assuming that contour grouping can be modulated top-down on the level of sensory or perceptual processing. We here suggest that top-down attention towards contours, as seen in θ_1_ PLV, operates on a local scale within earlier visual cortex. Based on the results of animal studies, Womelsdorf et al. [42] argued that long-range θ synchrony can directly impact single neuron effectiveness by modulating interneuron inhibition within the targeted brain regions. This fact is especially interesting when considering the role that inhibitory interneurons play in primary visual cortex V1 [48]. Several studies revealed that V1 interneurons are critically involved in shaping the receptive field properties of a neuron [49]–[52]. For example, Sato et al. [51] found that blocking activity of inhibitory interneurons between orientation-selective monkey V1 neurons reduced the sharpness of their tuning profiles so that stimuli with non-optimal orientations could evoke a response in that neuron. Such a change in feature selectivity would have direct consequences for the emergence of local association fields. Consider that local associations emerge if neurons with a given orientation preference co-activate neighboring neurons with a similar orientation preference. If neurons were now less sharply tuned to one orientation, then also sub-optimally oriented input would activate the neuron and its neighbors. Consequently, association fields would also occur for less smoothly aligned local elements, giving rise to the percept of a contour. Our S2 results fully comply with this interpretation. If a contour array was expected in a S2 display, a reduced θ_1_ phase-locking was observed compared to trials where a non-contour is expected. The PLV decrease might index a reduced interneuronal inhibition that produces a reduced orientation selectivity of early visual cortex neurons, which in turn should favor the establishment of local association fields. The viewpoint outlined above might also explain why no post-stimulus increase in β power was observed for S2 contour arrays. To the extent that preparatory θ_1_ synchronization favors the emergence of association fields, the percept of a contour might occur more readily and might draw on less neural resources. Thus, a β power increase does not show up for S2 contour arrays.

Alternatively, top-down control could also affect contour integration on higher stages of visual processing. Watt et al. [53] distinguish two operations in contour detection tasks: Linking of element to its neighbors, and assessing the goodness of the resultant contour. Linking occurs between each two elements of a display that have a compatible orientation and position. But the resultant contour would produce a positive response only if it matches a pre-defined contour prototype. It is conceivable that the described β power modulation reflects differences related to the contour description, rather than differences in contour linking per se. This alternative explanation cannot be fully excluded at present. However, the early onset and the occipital topography of the β_1_ effect support our interpretation that the difference is sensory in nature. Perceptual decisions based on object descriptions are thought to depend on more posterior (middle temporal) brain activity and should occur later in the processing stream [54]–[55]. The idea that top-down modulation affects early visual processing is also more in line with Li et al.’s [8] study where firing rates of monkey V1 neurons were investigated. It seems therefore appropriate to assume an early locus of top-down modulation in contour integration.

It should finally be noted that contextual modulation of neural responses in visual cortex does not necessarily involve horizontal interactions on the same level of the neural architecture [56]–[57]. For example, it was found that neurons coding which of two intersecting objects ‘owns’ a given outline integrate contextual information from larger and smaller objects with the same speed. This is incompatible with a horizontal propagation approach because greater distances between the receptive fields of neurons coding the border and context information should produce larger delays in propagation [58]. The fact that contextual modulation can occur without horizontal connections does not invalidate the arguments made in the present work. However, it shows that proposed mechanism for the attentional modulation of contour processing cannot readily be transferred to other domains of perceptual grouping.

We propose that the pre-stimulus θ synchronization observed in our study is related to top-down modulating activity in early visual areas. By decreasing the activity of inhibitory interneurons, the orientation-selectivity of visual neurons might be down-regulated allowing association fields to emerge for a broader range of contours that form a less smooth path. Although this explanation is as yet speculative, it integrates the seemingly opposing viewpoints that contour grouping arises bottom up within early visual cortex while at the same time depends on top-down control [2], [8]. Our view thus suggests how attention can be administered to visual objects that are not defined by a location or by a common feature.

### Conclusions

In summary, we investigated oscillatory brain responses in a contour grouping task and found preparatory brain activity when subjects expected to see a contour in the upcoming stimulus, as well as reduced post-stimulus activity compared to trials where the contour was not expected. The result shows that contour grouping, commonly considered to be a bottom-up driven process, involves top-down control whenever relevant pre-stimulus information is available. It is proposed that neural responses to perceptual objects can be shaped top-down by up- or down-regulating lateral inhibition in early visual cortex.
